# Prevalence and risk factors of antimicrobial resistance patterns of *Staphylococcus* spp. and *E. coli* in rodents and shrews at human-animal interfaces in Chattogram, Bangladesh

**DOI:** 10.1371/journal.pone.0327857

**Published:** 2025-07-10

**Authors:** Md. Aftabuddin Rumi, Pronesh Dutta, Monjurul Islam, Md Abu Sayeed, Md Kaisar Rahman, Md Helal Uddin, Shariful Islam, Ricardo J. Soares Magalhaes, Jonathan H. Epstein, Mohammad Mahmudul Hassan, Ariful Islam

**Affiliations:** 1 Faculty of Veterinary Medicine, Chattogram Veterinary and Animal Sciences University, Chattogram, Bangladesh; 2 Institute of Epidemiology, Disease Control and Research (IEDCR), Dhaka, Bangladesh; 3 National Centre for Epidemiology and Population Health (NCEPH), The Australian National University, Canberra, Australia; 4 School of Veterinary Medicine, Texas Tech University, Amarillo, United States of America; 5 Global Change Center, Virginia Tech, Blacksburg, Virginia, United States of America; 6 Queensland Alliance for One Health Sciences, School of Veterinary Science, The University of Queensland, Gatton, Australia; 7 One Health Science, United States of America; 8 Biosecurity Research Program and Training Centre, Gulbali Institute, Charles Sturt University, Wagga Wagga, New South Wales, Australia; 9 Training Hub Promoting Regional Industry and Innovation in Virology and Epidemiology, Gulbali Institute, Charles Sturt University, Wagga Wagga, New South Wales, Australia; Fayetteville State University, UNITED STATES OF AMERICA

## Abstract

Antimicrobial resistance (AMR) poses a significant threat to human and animal health worldwide. Wild rodents and shrews may serve as bioindicators of environmental health. They may serve as a potential source of the transmission of AMR bacterial infections to humans and domestic animals, despite not directly consuming antibiotics. We conducted a cross-sectional study aimed to estimate the prevalence and factors associated with the AMR patterns in *Staphylococcus* spp. and *Escherichia coli* (*E. coli*) isolated from rodents and shrews. We trapped and collected throat and rectal/urine swab samples from 200 wild rodents (n = 115) and house shrews (n = 85) across different locations in Chattogram, Bangladesh. The collected samples were then evaluated for the isolation of both bacterial organisms using culturing and biochemical properties. We performed culture sensitivity (CS) tests of the isolates using the Kirby-Bauer disc diffusion method for 14 antimicrobials. The overall prevalence of *Staphylococcus* spp. was 26.5% (95% CI: 0.20–0.33; n = 53), and *E. coli* was 56% (95% CI: 0.49–0.63; n = 112) in the sampled rodents and Asian house shrews. *Staphylococcus* spp. isolates were 100% resistant to oxacillin, oxytetracycline, metronidazole, and cefixime. Again, *E. coli* isolates were 100% resistant to metronidazole followed by ampicillin and cefixime (98.0%), sulfamethoxazole + trimethoprim (97.0%), amoxicillin and doxycycline (96.0%), streptomycin (95.0%). Only gentamycin was sensitive against both bacterial isolates. Statistical modeling revealed a higher risk of resistant bacterial infection in rodents from agricultural interfaces compared to other habitats. Rodents and Asian house shrews with poor body condition were more prone to resistant *Staphylococcus* spp. infection, while rodents were more susceptible to resistant *E. coli* infection. Our findings indicate a significant prevalence of AMR *Staphylococcus* spp. and *E. coli* in urban rodents and house shrews, suggesting their potential role as reservoirs and disseminators of AMR, hence posing a risk to human and animal health.

## Introduction

Rodents, comprising almost 40 percent of all species of warm-blooded animal and include a range of species from mice, rats, squirrels, prairie dogs, porcupines, beavers, guinea pigs, hamsters, gerbils and capybaras [1]. Globally there are 5,450 recognized species of small mammals in both wild and domestic settings [1], and 127 species reported in Bangladesh [2]. Among these, small wild mammals such as black rats*,* bandicoot rats, Indian mole-rat, house mice, and Asian house shrews are frequently found in Bangladesh [3]. These animals often come into close contact with human dwellings for food and habitat purposes [4], increasing the likelihood of human-animal interactions. These interactions bring with them the risk of zoonotic disease transmission, especially in rapidly growing populations.

The increasing human population and resulting fragmentation of natural ecosystems force wild animals to live in close proximity to humans and livestock, further raising the risk of zoonotic disease transmission and threatening public health [5]. Rodents have historically been associated with numerous epidemics, serving as potential sources of over 88 zoonotic diseases [6,7]. Rodents are the vertebrate reservoirs of infectious diseases of public health significance such as *Yersinia pestis*, which causes bubonic plague, leptospirosis, hantavirus, as well as other diseases like typhus, Weil’s illness, toxoplasmosis, and trichinosis [8]. This connection between rodents and public health concerns extends to major zoonotic foodborne illness as well, particularly through peri domestic rodents [9]. The transmission of foodborne pathogens highlights the importance of understanding the role rodents play in zoonotic diseases.

Rodents are reported reservoirs of several pathogenic bacteria including *Campylobacter* spp. [10], *Clostridium* spp. [11], *Coxiella burnetii* [12], *Escherichia coli* (*E. coli*) [13], *Staphylococcus* spp. [14], *Salmonella* spp. [15]. More recently, a highly contagious virus responsible for severe diarrhea in children, was identified in rodents and shrews in Bangladesh [16]. Again, zoonotic parasites such as *Hymenolepis* spp. and *Capillaria* spp. have been found in the gastrointestinal tract of the free-ranging Asian house shrews in Bangladesh [17]. These findings suggest that small mammals may play a significant role in the spread of zoonotic pathogens, including those that are part of their normal flora. Among the organisms reported to be carried by small mammals, *Staphylococcus* spp., and *E. coli* are part of their normal flora [6], but some strains can cause serious illness in humans and animals. For instance, *Staphylococcus aureus (S. aureus)* is a major cause of various human illnesses, such as impetigo, abscesses, endocarditis, and foodborne illness [18]. *E. coli* is known to cause cholecystitis, bacteremia, cholangitis, urinary tract infection (UTI), neonatal meningitis, pneumonia in humans [19]. Moreover, Shiga-toxin-producing *E. coli* (STEC) is a major foodborne health threat that causes mild diarrhea, hemorrhagic colitis and potentially fatal hemolytic uremic syndrome [20]. These pathogenic strains of *Staphylococcus* spp., and *E. coli* can be dangerous not only to humans but also to animals, making their control crucial to refraining from public health risk.

The growing concern for these pathogens is spanning around the development of AMR. Earlier, study shows that most of the infections with *S. aureu*s has developed resistance to methicillin, leading to the emergence of Methicillin-Resistant *Staphylococcus aureus* (MRSA), which is harder to treat with standard antibiotics and poses a greater public health threat [21]. In addition, high levels of *E. coli* resistance have been reported in humans, livestock species, and small mammals [6,22,23]. Resistant bacteria are also found with high prevalent among wild rodents living in closeness to livestock and human residence [24,25]. This suggests that the proximity of wild animals to human activity, especially livestock farming, plays a role in the persistence and spread of AMR.

The relationship between anthropogenic factors and the prevalence of AMR is becoming increasingly evident. For instance, AMR *E. coli* isolates from the excretion of small mammals living in livestock farms show significant resistance to ampicillin, amoxicillin-clavulanic acid and tetracycline, which is higher than the small mammals sampled from other sites [24]. Moreover, animals living closer to human populations, and livestock farms exhibit higher levels of AMR compared to animals living in natural environment with no or less human contact [26,27]. These findings indicate that human and livestock activities may be contributing to the development and persistence of AMR in wild mammals. In order to better understand this relationship, eco-epidemiological research activities provide an approach that integrates ecological influences, societal, and population-based perspectives to study the environmental risk factors.

Recent studies have started to consider wildlife including small mammals such as rodents and shrews as possible bioindicators or sentinels for AMR, besides zoonotic disease transmission [28–30].Resistance to antibiotics is widespread in some wild rodent populations living in areas with no known presentation to antibiotics [31], whereas, sampling in natural environmental regions did not detect widespread resistant bacteria in wild rodents [27].

Given the lack of such studies in Bangladesh, this research aims to determine the prevalence and risk factors of AMR patterns in *Staphylococcus* spp. and *E. coli* recovered from rodents and house shrews at the human-livestock interfaces in Chattogram, Bangladesh. This study will fill an important gap in understanding how small mammals serve as reservoirs and spreaders of AMR thus posing a threat to human and animal health.

## Materials and methods

### Ethical approval

The study protocol was reviewed and approved by Chattogram Veterinary and Animal Sciences University (CVASU)-Animal Experimentation Ethics Committee (AEEC), Chattogram, Bangladesh. The CVASU-AEEC approval number of this project was: (CVASU/Dir (R& E) AEEC/2015/751, Dated: 05/09/2016).

### Study location and duration

**Fig 1:**
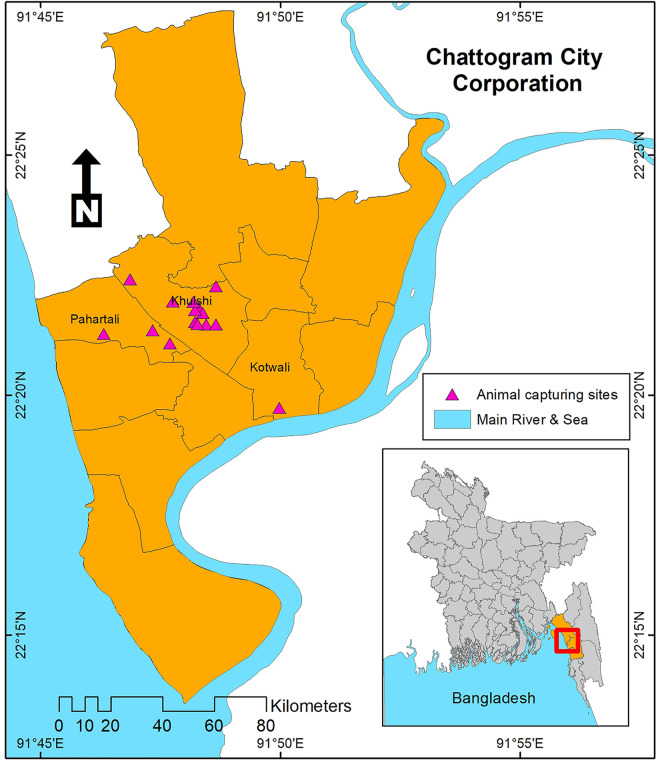
Capturing sites of the rodents and Asian house shrews in Bangladesh. The inset shows the study district in Bangladesh. The triangular shape represents the capturing sites. The map was created using a shapefile from the freely accessible GADM database (GADM; www.gadm.org) [34].

We conducted a cross-sectional study from January to June 2017 in Chattogram district, Bangladesh (Fig 1). Chattogram is a district (small administrative region) with diverse geography including forest areas and hills with an abundance of many wild small mammals like rodents, Asian house shrews, and many other species [32]. We purposively targeted those locations which could constitute human-livestock interfaces or gradients where antimicrobial-resistant bacteria can be present in the environment due to nearby antimicrobial use in hospital settings (Chattogram Medical College Hospital, Chattogram General Hospital, Chattogram Diabetic General Hospital, Bangabandhu Memorial Hospital, Shahedul Alam Quadery Teaching Veterinary Hospital) and the availability of the antimicrobial residues in the surrounding environments (Sagorika cattle market) [33].

### Sample size calculation and sampling

Simple random sampling was used for the sample collection, and the sample size was estimated by the formula described by [35]. As there were no existing population estimates for rodents and Asian house shrews in the Chattogram region, we defined our sampling frame based on categorized sites: (i) agricultural sites with low human density and minimal infrastructure, (ii) human dwellings with high population density, and (iii) marketplaces with commercial infrastructure [16,36]. We estimated a total sample size of 384, considering 50% expected prevalence, 5% precision, and a 95% confidence interval (CI). Although, within the sampling sites, trapping locations were randomly selected to ensure representation across different environments, but due to our trapping success, time constrains, and resource limitations, we were able to capture only 200 animals for this study.

### Rodent and shrew capturing, sample collection, processing, and storage

Locally made steel wire traps baited with ghee-smeared biscuits and dried fish were used to capture rodents and Asian house shrews. Traps were set daily at dusk and collected at dawn the following day [16] labeled with the trapping date and species. To account for pseudo replication in our rodent and shrew capture data, we employed mark–recapture techniques to reliably identify previously captured individuals. We used a combination of nontoxic permanent dye on fur and feet toe, and shaving patterns to mark animals upon first capture. These methods allowed us to visually identify recaptured individuals and ensure they were not counted as independent observations. Additionally, traps were placed at different locations rather than the same sites to reduce the likelihood of recapturing the same individuals in the same area. We anesthetized the captured rodent and Asian house shrews using were inhalant anesthetic; isoflurane following established procedures [17]. The rodent and Asian house shrew species were identified based on morphometric and photographic data published in a scientific journal [37,38]. Oral and rectal/urine swab samples were collected from each subject using sterilized swab sticks and placed in sterile vials containing 10 ml Buffer Peptone Water Media (OXOID, UK). Vials were labeled with a unique identification number and sample type. After collection, samples were immediately stored in cool boxes (4°C), transported to the Poultry Research and Training Centre (PRTC), CVASU laboratory, and stored in a refrigerator at −20°C until further laboratory evaluation.

### Data collection

Besides laboratory data, we also gathered ecological and environmental data through direct observation at animal trapping locations with the help of a questionnaire checklist. The observational records were centered on environmental settings of rodent habitats, human residences, and likely human-rodent interactions. Geo-coordinates of the trap locations were also logged through geographic information system (GIS) machine. Morphometric and demographic information of trapped rodents and shrew’s species, sex, age category, body condition score, and overall health status were recorded at the time of sample collection. We did not record any personal and human-identifiable information at all during the research. It was an observational and ecological survey of free-living wild animals in domestic environments, agricultural farms, and markets. Informed verbal permission from household owners was obtained prior to placing traps close to their residence. The field team documented these permissions in the filed data sheets. Given the non-invasive nature of the study and the absence of direct human participation or identifiable data collection, formal human ethics approval and written consent were not required, as confirmed by the ethics committee. The committee concluded that verbal rather than written consent was appropriate as the risk was minimal.

### Laboratory evaluation

#### Isolation and identification of bacteria.

In the laboratory, we enriched the samples in Buffer Peptone media to induce the growth of bacteria, including *Staphylococcu*s spp. and *E. coli*. Individual samples were streaked on blood agar followed by Mannitol Salt Agar for culturing *Staphylococcu*s spp. [14] and MacConky Agar followed by Eosin-Methylene Blue (EMB) Agar for *E. coli* [39]. All the samples of agar plate were then incubated at 36^º^C temperature for 24 hours. After incubation, we studied the morphological and colony characteristics of the bacterial cultures and selected representative colonies for Gram’s staining. Finally, we confirmed the isolated bacteria using biochemical tests: coagulase and catalase tests for *Staphylococcu*s spp. [40,41], and indole and carbohydrate fermentation tests for *E. coli* [39].

#### Antibiogram study.

Antimicrobial susceptibility testing was performed on Muller-Hinton Agar (Liofilchem, Italy) using the Kerby-Bauer micro-disc diffusion method [42]. Fourteen commonly used antimicrobials in Bangladesh for both human and animal health were selected: sulphamethoxazole + trimethoprim, ampicillin, ceftriaxone, oxacillin, azithromycin, ciprofloxacin, streptomycin, oxytetracycline, cefixime, amoxicillin, erythromycin, gentamycin, metronidazole, and doxycycline [43]. In brief, a bacterial turbidity equivalent to 0.5 McFarland standard was prepared for each positive isolate followed by steaking onto Mueller Hinton agar plate, and antimicrobial discs were placed centrally using an antimicrobial disc dispenser [42]. Plates were incubated at 37°C for 24 hours and then observed for zones of inhibition, which were measured in millimeters using digital slide calipers. Isolates were classified as sensitive (S), intermediate sensitive (I), or resistant (R) to the tested antimicrobials based on CLSI 2007 guidelines and the manufacturer’s (OXOID, UK) interpretation criteria for *Staphylococcus* spp.*, and E. coli* [44].

#### Statistical analysis.

All the biological, laboratory, geocoordinate and ecological data were recorded into the Microsoft Office Excel-2017 spreadsheet and later imported into R studio 2024.04.2 for analysis [45]. Descriptive statistics were performed to summarize the antibiogram pattern of *Staphylococcus* spp. and *E. coli* isolated from oral and rectal/urine swabs from the sampled rodents and Asian house shrews and expressed as a frequency and percentage. Results were expressed as ranges with 95% confidence intervals (CI). We used a chi-square test for independence (α ≤ 0.05) to identify associations between antimicrobial resistance prevalence and factors including age, sex, body condition score (BCS), species, and habitat. A multivariable logistic regression model was employed to identify significant factors associated with the bacterial AMR infection. Finally, we used marginal mean analysis of explanatory variables to determine the predictive probability of AMR positivity [46].

## Results

### Overall prevalence of *Staphylococcus* spp. and *E. coli*

We captured a total of 200 rodents and shrews at market, agriculture, livestock farms and human dwelling intercedes in Chattogram City during the study period. Among them, 115 were rats (*Rattus rattus*, *Bandicota bengalensis*, and *Bandicoot indica*), house mice (*Mus musculus)*, and 85 were Asian house shrew (*Suncus murinus*). Among the captured rodents, 54 subjects were male and 61 were female, on the other hand, among the Asian house shrew, 42 were male and 43 were female. Most of the rodents and shrews were adults and few (10 rodents and 11 Asian house shrews) were juvenile. (**Table 1**).

**Table 1:** Demographic information of sampled rodents and Asian house shrews in Chattogram, Bangladesh.

Variable	Category	Rodent (n = 115)	Asian house Shrew (n = 85)
Sex	Male	54	42
	Female	61	43
Age	Juvenile	10	11
	Adult	105	74
BCS	Poor	17	14
	Good	98	71
Sampling interface	Agriculture side	15	6
	Human resident	60	52
	Marketplace	40	27

n = Number of animals.

**Fig 2:**
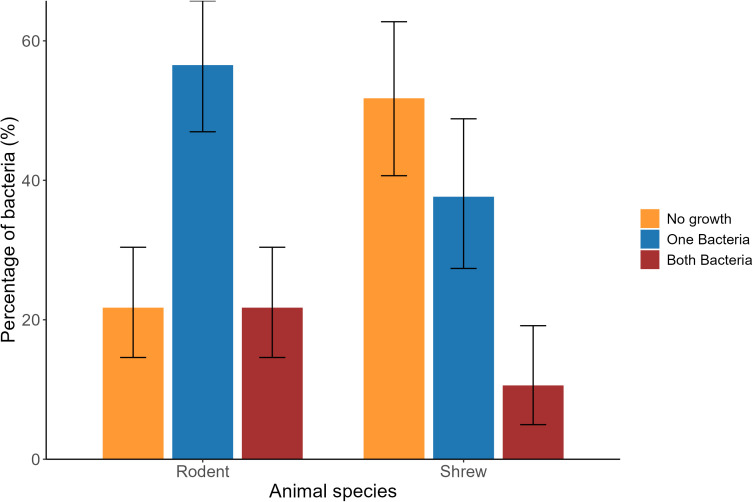
Percentage of identified bacteria in rodents and shrews in the study sites with 95% confidence interval.

In this study, we isolated 53 *Staphylococcus* spp. from the oral swab, and 112 *E. coli* from rectal and urine swabs. The overall prevalence of *Staphylococcus* spp. and *E. coli* was 26.5% (95% CI: 20.52–33.19) and 56% (95% CI: 48.82–62.99), respectively in captured animals. Additionally, 17.0% of total animal samples tested positive for both bacterial isolates. In between the animal taxa, 21.7% of rodent samples and 10.6% of Asian house shrew samples were positive for both bacteria (Fig 2). Among the identified bacteria, all isolates of *E. coli* and *Staphylococcus* spp. were shown AMR characteristics to at least one antimicrobial.

#### Descriptive statistics for AMR in *Staphylococcus spp.* and *E. coli.*

The presence of resistant *E. coli* bacterial infection was significantly higher in animals captured at the agricultural interface (71.4%), followed by the market interface (64.2%) and human dwelling (48.2%) (Table 2). A similar pattern was observed for resistant *Staphylococcus* spp. infection in these habitats. Rodents (68.7%) had a notably higher percentage of resistant *E. coli* presence compared to Asian house shrews (38.8%), and the same trend was seen for resistant *Staphylococcus* spp., with rodents (31.3%) having a higher prevalence than Asian house shrews (20.0%). Sex and age of the animals did not show significant differences between categories. However, animals with a poor BCS had a higher prevalence for both *Staphylococcus* spp. (54.8%) and *E. coli* (64.5%).

**Table 2:** Proportion of identified AMR *Staphylococcu*s spp. and *E. coli* bacteria in the rodents and Asian house shrews in Chattogram.

Factor	Category (N)	*Staphylococcus* spp.			*E. coli*		
		Positive, n (%)	95% CI	p-value	Positive, n (%)	95% CI	p-value
Habitat	Human dwellings interface (112)	22 (19.6)	12.74–28.21	0.03	54 (48.2)	38.67–57.85	0.04
	Market interface (67)	22 (32.8)	21.85–45.4		43 (64.2)	51.53–75.53	
	Agriculture interface (21)	9 (42.9)	21.82–65.98		15 (71.4)	47.82–88.72	
Taxa	Rodents (115)	36 (31.3)	22.98–40.62	0.07	79 (68.7)	59.38–77.02	<0.01
	Asian house Shrews (85)	17 (20.0)	12.1–30.08		33 (38.8)	28.44–50.01	
Sex	Male (96)	26 (27.1)	18.52 −37.11	0.86	59 (61.5)	50.97–71.22	0.14
	Female (104)	27 (26.0)	17.86–35.48		53 (51.0)	40.97–60.9	
Age	Juvenile (21)	5 (23.8)	8.22–47.17	0.77	13 (61.9)	38.44–81.89	0.56
	Adult (179)	48 (26.8)	20.48–33.94		99 (55.3)	47.71–62.73	
BCS	Good (169)	36 (21.3)	15.39–28.25	<0.01	92 (54.4)	46.61–62.1	0.29
	Poor (31)	17 (54.8)	36.03–72.68		20 (64.5)	45.37–80.77	

N = number of animals, n = number of positive, % = proportion, CI = Confidence Interval.

#### Risk factors for AMR in *Staphylococcus* spp. and *E. coli.*

**Fig 3:**
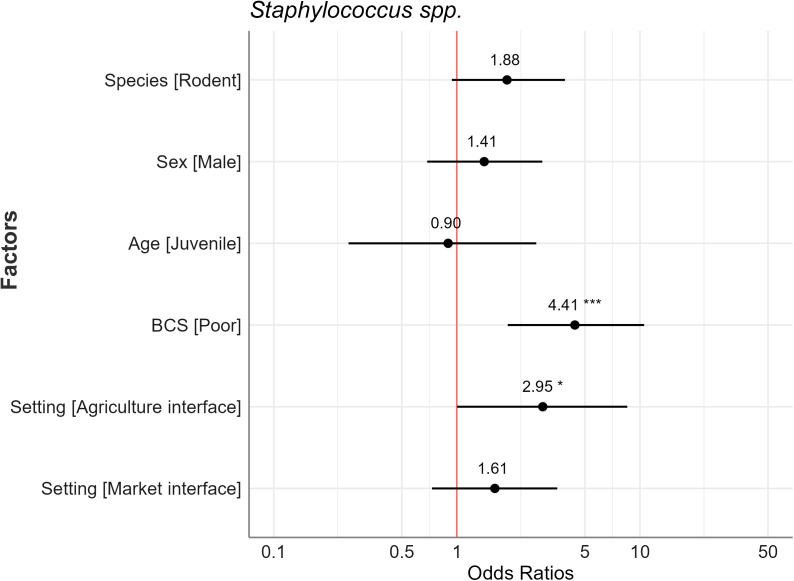
Forest plot of odds ratios for *Staphylococcus* spp. compared to the reference category for each independent variable (intercept and reference category not shown) with 95% confidence intervals and significance markers (*). The red line at the vertical intercept represents no effect (where the odds ratio equals 1 on the x-axis).

**Fig 4:**
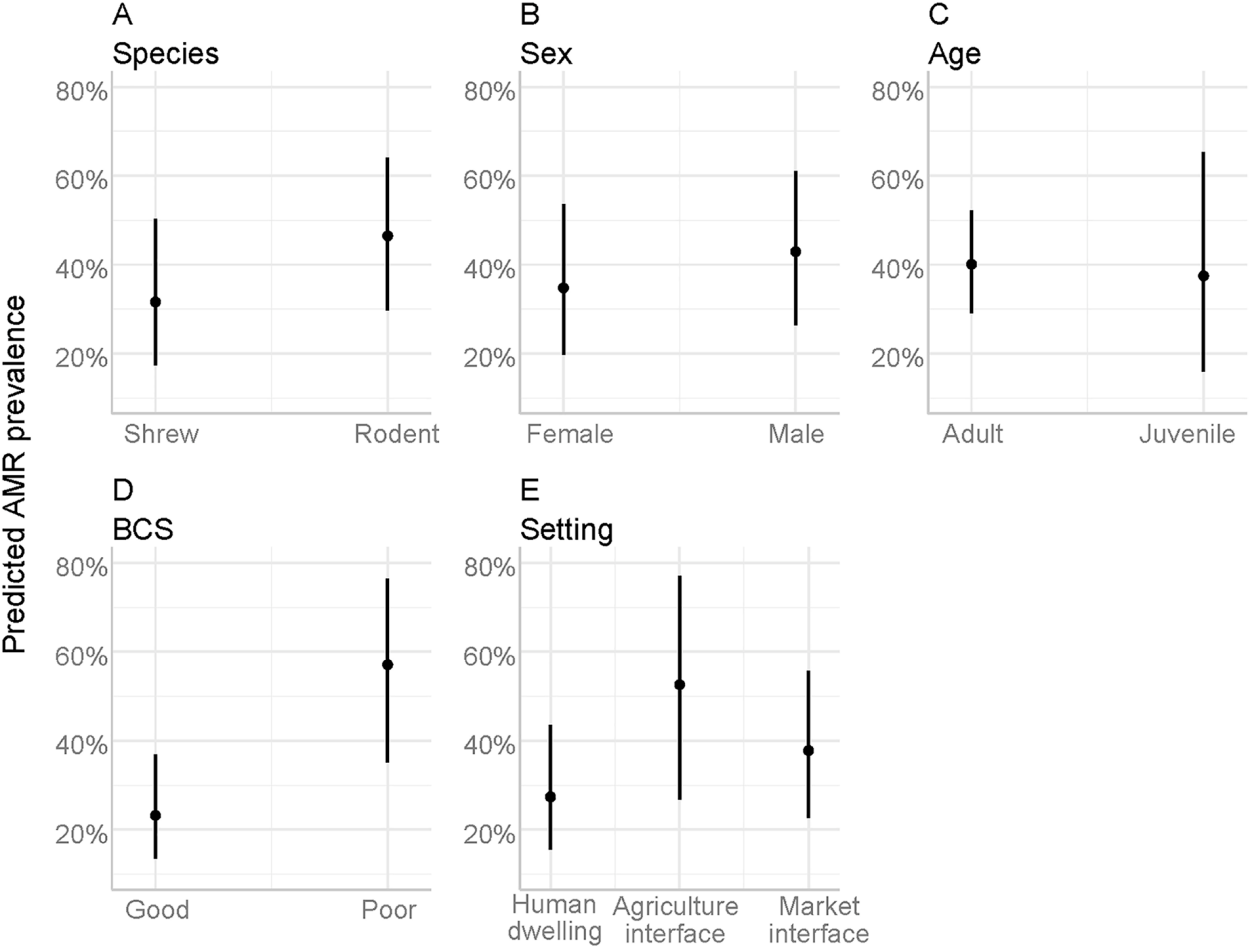
Estimated marginal means and 95% confidence intervals for *Staphylococcus* spp. across independent variables as a function of their respective explanatory variables.

In multivariable logistic regression, two factors had a significant association with resistant *Staphylococcus* spp. presence and three factors were significant for resistant *E. coli* presence. Captured animals in the poor BCS category were 4.41 times more likely to be infected with *Staphylococcus* spp. compared to those in the good category (Fig 3). The marginal mean graph also showed the predicted prevalence at 58.0% for poor (BCS) compared to 21.0% for good (BCS) (Fig 4).

**Fig 5:**
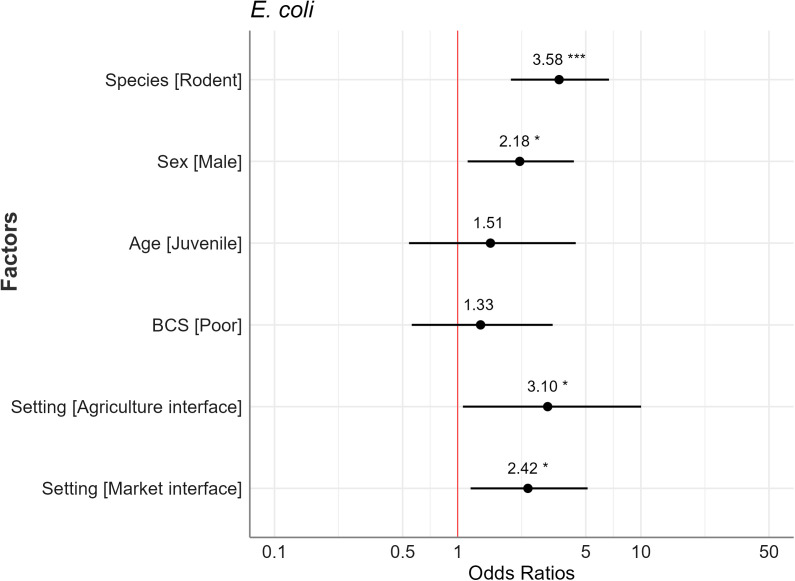
Forest plot of odds ratios for *E. coli* compared to the reference category for each independent variable (intercept and reference category not shown) with 95% confidence intervals and significance markers (*). The red line at the vertical intercept represents no effect (where the odds ratio equals 1 on the x-axis).

**Fig 6:**
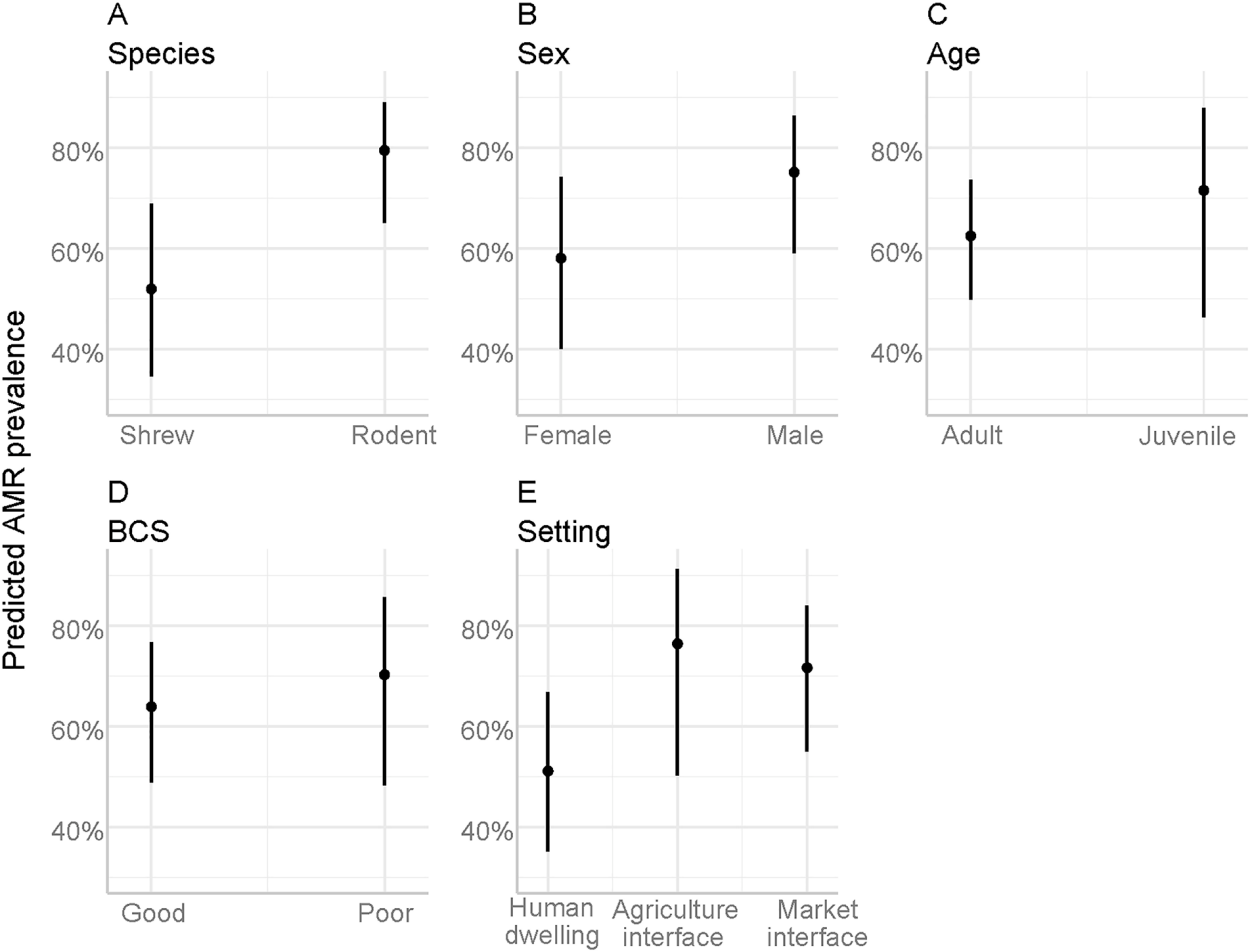
Estimated marginal means and 95% confidence intervals for *E. coli* across independent variables as a function of their respective explanatory variables.

Animals captured in the agriculture interface had 2.95 times higher odds of being AMR-positive for *Staphylococcus* spp. compared to those captured in the human dwelling interface (Fig 5). For *E. coli*, the odd was 3.10 times for the agriculture interface and 2.42 times for the market interface compared to the human dwelling. The predicted probability of resistant *E. coli* for the agriculture interface was 76.0%, for the market interface was 72.0%, and human dwelling interface was 51% (Fig 6). Rodents had 3.58 times higher odds than Asian house shrews, and male animals had 2.18 times higher odds compared to females of being positive for resistant *E. coli* bacteria.

### Antimicrobial sensitivity pattern of isolated bacteria

**Fig 7:**
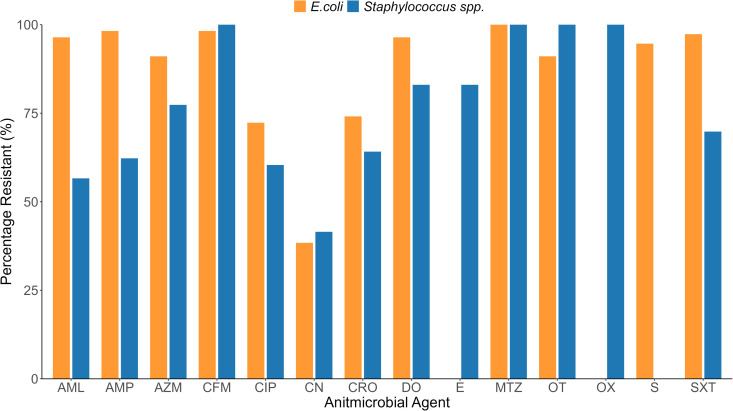
Resistance pattern of *Staphylococcus* spp. and *E. coli* isolated from the rodents and Asian house shrews. Note: SXT = Sulphamethoxazole+Trimethoprime, AMP = Ampicillin, CRO = Ceftriaxone, OX = Oxacillin, AZM = Azithromycin, CIP = Ciprofloxacin, S = Streptomycin, DO = Doxycycline, CFM = Cefixime, AML = Amoxicillin, E = Erythromycin, CN = Gentamycin, MTZ = Metronidazole, OT = Oxytetracycline.

Each of the isolated bacteria was subjected to the CS test against 13 (against *Staphylococcus* spp.) and 12 (against *E. coli*) antibiotics which are commonly used in both human and animal health in Bangladesh. We presented the test results according to animal species. In the CS test of the *Staphylococcus spp* isolates against some antimicrobials such as oxacillin, cefixime, metronidazole, and oxytetracycline were observed 100% resistance, followed by doxycycline, erythromycin, and azithromycin. The other antimicrobials including sulphamethoxazole **+** trimethoprim, ceftriaxone, ampicillin, ciprofloxacin, and amoxicillin showed moderate resistance patterns. Only Gentamycin presented significant sensitivity in the CS test of *Staphylococcus* spp. On the other hand, *E. coli* isolates were detected with 100% resistance against Metronidazole and 62.0% sensitivity to Gentamycin. The other antimicrobials also showed a higher level (72–98%) of resistance patterns of *E. coli* (Fig 7).

**Fig 8:**
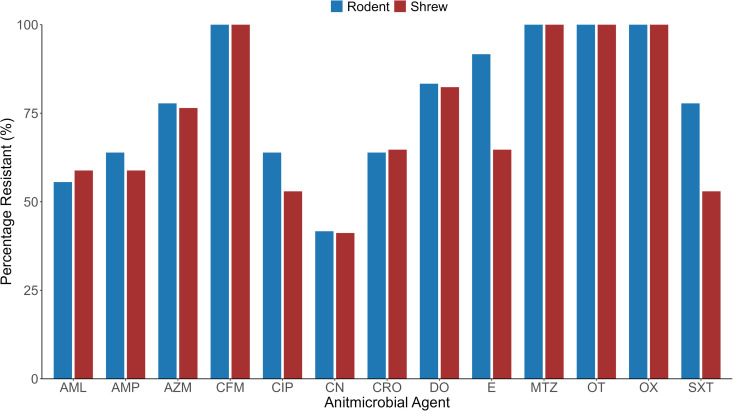
Resistance pattern of *Staphylococcus* spp. isolated from the rodents and Asian house shrews. Note: SXT = Sulphamethoxazole + Trimethoprime, AMP = Ampicillin, CRO = Ceftriaxone, OX = Oxacillin, AZM = Azithromycin, CIP = Ciprofloxacin, DO = Doxycycline, CFM = Cefixime, AML = Amoxicillin, E = Erythromycin, CN = Gentamycin, MTZ = Metronidazole, OT = Oxytetracycline.

According to animal species, *Staphylococcus* spp. isolates showed 100% resistance against oxacillin, cefixime, metronidazole, and oxytetracycline in both animal species. Among the rest of the antibiotics, *Staphylococcus* spp. isolates showed resistance against erythromycin, followed by sulphamethoxazole **+** trimethoprim, doxycycline, azithromycin, ceftriaxone, ciprofloxacin, and ampicillin. *Staphylococcus* spp. isolates in rodents and Asian house shrews were observed a significant level of sensitivity against only gentamycin (Fig 8).

**Fig 9:**
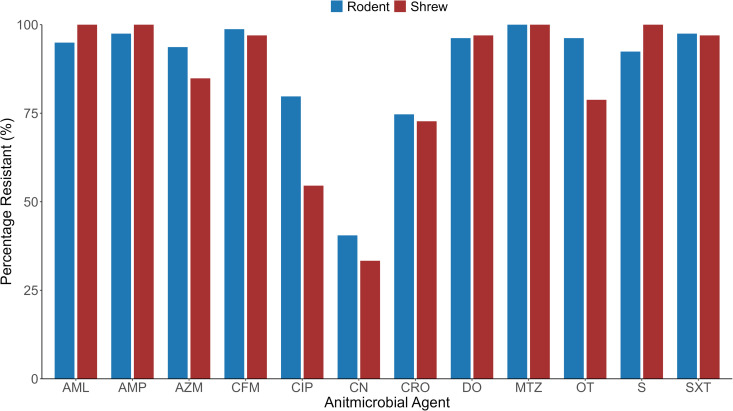
Resistance pattern of *E. coli* isolated from the rodents and Asian house shrews. Note: SXT=Sulphamethoxazole + Trimethoprime, AMP=Ampicillin, CRO=Ceftriaxone, AZM= Azithromycin, CIP=Ciprofloxacin, S=Streptomycin, DO=Doxycycline, CFM=Cefixime, AML=Amoxicillin, CN=Gentamycin, MTZ=Metronidazole, OT=Oxytetracycline.

On the other hand, *E. coli* isolates were shown more resistance against antimicrobial agents in rodents than Asian house shrews. In rodents, *E. coli* isolates showed 100% resistance against metronidazole in both animal species. Besides, in rodents *E. coli* isolates showed higher resistance against other antimicrobial agents such as ceftriaxone (99%), sulphamethoxazole + trimethoprim, and ampicillin (97%), doxycycline and oxytetracycline (96%), and 80–95% with amoxicillin, azithromycin, streptomycin, ciprofloxacin. On the other hand, *E. coli* isolates showed 100% resistance against ampicillin, amoxicillin, and streptomycin, followed by sulphamethoxazole + trimethoprim, doxycycline, and cefixime (97%), azithromycin (85%). In our study, we found only gentamycin had effectiveness against the *E. coli* of rodents and Asian house shrews (Fig 9).

## Discussion

Antimicrobial resistance is a threatening global health issue, not only for human health but also for animal health. Some microorganisms, such as commensal Enterococci and *E. coli*, have been effective markers for assessing the extent of resistance across different populations and the spread of resistant bacteria between ecosystems. Nevertheless, AMR research in wild animals and their environment is very limited in Bangladesh. Intending to conduct AMR research on wildlife, the present study brings out the first eco-epidemiological investigation in Bangladesh.

We found the presence of *Staphylococcus* spp. and *E. coli* in the fecal and oral swabs of the free range rodents in the human-wildlife interface, which indicates that wild animals can get bacterial infection through contact with human or other domestic animals and environmental contamination [29,47,48]. Our study area was in the southern part of Bangladesh, which is very close to the hilly area with forests, and also a reservoir of many wild animal species with a close relationship among wild animals, humans, and livestock in this environment [49]. Besides, small mammals (rats, house mice, and Asian house shrews) live closer to humans and livestock for food and nesting and act as vectors for disease transmission as well as a pests [50].

The presence of *Staphylococcus* spp. in rodents in our study is similar to the findings of [51,52] and comparable with the other studies in different countries, where *S. aureus* was identified in 15.3–74.3% of rodents [14,53,54]. Among all of the species of *Staphylococcus* spp.; *S. aureus* is highly pathogenic and exists in the air, water, dirt, and excretions of the hospital patients [55], whereas *S. epidermidis* is less pathogenic and may live on the body surfaces of the animal as a normal flora [56]. The presences of *Staphylococcus* spp. in all three small mammal species in the present study have a lower level than in a previous study in China where it was reported at 75% in shrew [57].

In our study, *E. coli* was identified with higher percentages in wild rodents in the Chattogram district, similar to the findings of [30]. However, a higher presence of *E. coli* in wild rodents has been reported in several studies in different countries varying from 14.8% – 90% [39,58–60]. This result indicates that *E. coli* is more frequent in wild animals that are roaming freely in both wild and human-resident areas [61]. It may be due to environmental pollution done by humans and the shedding of bacteria by a human to the environment, which may transmit bacteria to small mammals [62]. However, most *E. coli* strains are commensals in a variety of mammalian hosts, but pathogenic or enterotoxigenic strains are known to exist [63,64], which may have an epidemiological contribution to the spread of resistant bacteria into other animals and humans.

The close vicinity of human dwellings to the wildlife habitat has an essential role in pathogen transmission in free-ranging wildlife and its environment [65,66]. The urban landscape, where a wild animal is often exposed to anthropogenic disturbance shows more frequency in pathogen contamination. An average of 12% *E. coli* infection was found in Spain in rural rodents, while 47.4% was recorded in an urban area close to human settlement. Therefore, the influence of human disturbances and behavior is the possible cause of higher prevalence in the studied roosting site [65]. Similarly, Jardine, Janecko [67] conducted a study on rodents near the pig farm in Canada, where a higher prevalence of *E. coli* (24.95%) was reported. Pathogenic *E. coli* outbreaks in agricultural production have been linked to wild animals, however, understanding the genetics of *E. coli* in wild animals remains a significant knowledge gap [61]. The presence of bacterial load is higher in all of these small mammals due to their feeding on garbage and contaminated food materials and inhabitants close to that area where bacterial load is higher in nature [68]. The resistance pattern of *Staphylococcus* spp. against some common antimicrobials is not frequently reported in previous studies of antimicrobial research in wild small mammals [69]. The majority of *Staphylococcus* spp. in wild small mammals were reported comparatively lower than our study prevalence [30,31]. Bacterial load for *E. coli* is significantly higher in different rat species than in house mice and Asian house shrews, which may be due to the close contact of rats with humans and the wild environment than the mice shrew. Overall rates of bacterial isolation in our study are comparatively lower than in a previous study on shrews in China, which may be due to variations in the location [57].

In the case of *Staphylococcus* spp. antimicrobial sensitivity test for 13 antibiotics, we found that rat and shrew possess 100% resistance against oxacillin, cefixime, metronidazole, and Oxytetracycline in contrast, isolates in mice additionally showed resistance against one more antibiotic; Erythromycin, which indicates that house Mice have a comparatively higher prevalence of resistance than different rat species and Asian house shrew. In the CS test result, *E. coli* isolates showed 100% resistance against Metronidazole antibiotics which is much higher than in previous studies on small mammals [70–72]. Another previous study on vole and mice in England showed that *E. coli* isolates are resistant (67–100%) to amoxicillin, tetracycline, trimethoprim, and cefuroxime which are almost similar to our study [73]. A survey of wild rodents reported that isolated *E. coli* have a multidrug resistance which findings are similar to our study [74,24]. In Canada, a study on rats and mice reported that isolated *E. coli* has a resistance (4.2–17%) against ampicillin, amoxicillin, cefoxitin, gentamycin, tetracycline, ceftiofur, streptomycin, which is much lower in comparison to our study findings [31]. Since rodents have significant opportunities to contact human sewage systems and garbage dumps in urban areas, they can get resistance *E. coli* infection from humans and their surrounding animals [75].

A previous report on wild mammals revealed that almost all the analyzed strains (97.7%) showed resistance/intermediate resistance to at least one class (β-lactams, tetracyclines, quinolones, aminoglycosides, sulphonamides, polypeptides, and phenicols) of antibiotics and the highest resistance values were observed for the tetracycline class which is similar to our study findings [76].

Overall, it indicates that mice had a comparatively higher number of resistant antibiotics for *Staphylococcus* spp. but in *E. coli* infection, it had relatively lower resistance against antibiotics. Besides, our study found a significant level of infection with *E. coli*, and *Staphylococcus* spp. in rodents, mice, and Asian house shrews with evidence which is an alarming issue for our country because this level of resistance in those species of wild animal species is not acceptable due to their disjuncture with an antimicrobial [74,24]. Further study should be done on this aspect to control the rate of resistance and prevent the spreading of this resistance to humans, animals, and the environment from those small mammal species.

This is the first report on the prevalence and antibiotic resistance patterns in *E. coli* and *Staphylococcus* spp. isolates on wild small mammals in Bangladesh. This study represents baseline data on how prevalent and antimicrobial resistance features in wild rodents and shrews, representing part of the environment in Bangladesh. Wild rodents may contribute to the spread of antibiotic resistance to other wildlife and environmental spheres through fecal contamination. Therefore, it is crucial to implement a comprehensive surveillance program for antimicrobial-resistant bacteria in wildlife, particularly in species residing near human habitats, for strengthening surveillance systems for food animals, food, and humans. This study was undertaken with a smaller sample than the statistically calculated sample size. The capturing process of rodents and shrews is time-consuming, and the success of the capture depends on the population density of the rodents and shrews, availability of the food sources, and seasonal variation. Another limitation was that we captured animals only from one district, primarily due to our time and resource constraints. We identified only two enteric bacteria and analyzed their antibiograms in the fecal samples of rodents and shrews. However, these two bacteria commonly infect humans and other mammals and are responsible for several illnesses. We didn’t detect the resistant genes of the identified bacteria, which was another limitation of our study, though research on AMR in rodents and shrews is limited in Bangladesh.

## Conclusion

The study demonstrated a high frequency of AMR *Staphylococcus* spp. and *E. coli* in a substantial proportion of fecal and oral samples from free-ranging rodents and Asian house shrews in Chattogram, Bangladesh. Notably, rodents and Asian house shrews captured at the agriculture interface and those with poor body condition more likely to carrying AMR bacteria. While all bacterial isolates displayed resistance to some antimicrobial agents, gentamycin remained effective against *Staphylococcus* spp. and *E. coli.* Our findings indicate that wild rodents and Asian house shrews may serve as reservoirs for zoonotic infections, hence posing a threat to human, animal, and environmental health. We recommend carrying out further studies that includes comprehensive sampling and molecular typing to identify resistant bacteria and genes within these populations, ascertain their origins, and clarify the role of these synanthropic rodents in the epidemiology of AMR and zoonotic transmission to humans. To mitigate the emergence of superbugs, essential AMR stewardship program must be implemented for the prudent use and disposal of antibiotics.

## Supporting information

S1 File Dataset. Snapshot of the sampled animals’ dataset.
(XLSX)

S2 File Questionnaire. Questionnaire used for collecting ecological data.
(DOCX)

S3 File PLOS Questionnaire. Questionnaire on inclusivity in global research.
(DOCX)
