# Supplementary material for: Prevalence and risk factors of antimicrobial resistance patterns of Staphylococcus spp. and E. coli in rodents and shrews at human-animal interfaces in Chattogram, Bangladesh
Source: PLoS One. 2025 Jul 10;20(7):e0327857. doi: 10.1371/journal.pone.0327857 (PMC12244769; doi:10.1371/journal.pone.0327857)
Supplement: S2 File — Questionnaire used for collecting ecological data. (DOCX) [file pone.0327857.s002.docx]

**Observational Questionnaire:** Prevalence and risk factors of antimicrobial resistance patterns of *Staphylococcus* spp. and *E. coli* in rodents and shrews at human-animal interfaces in Chattogram, Bangladesh.

**Section A: General information**

1. Date of Interview: ___ / ___ / ___
2. Name of the sampling site : ----------------------
3. GPS Coordinates: Latitude: ____________ Longitude: ____________

**Section B: Ecological and environmental information (Ecological observation)**

1. What types of buildings or infrastructure are present near the trapping site?

Hospitals or clinics/ Animal farms or livestock sheds/ markets/ Residential homes

1. What is the level of human activity in the trapping area?

Low / Moderate / High

1. What are the wild small mammals commonly seen around this location?
2. Rodents B) Asian house shrews C) Both, D) other wild animals------
3. What type of interface observed at the sampling site

A) Agriculture interface B) Human dwelling C) market interface

**Section C: Rodent Capture Data (Researcher only)**

1. Species Captured: *Rattus rattus* / *Bandicota bengalensis* / *Bandicoot indica* / *Mus musculus* / *Suncus murinus*
2. Sex: Male / Female
3. Age Group: Juvenile / Adult
4. Body Condition Score (BCS): Good / Fair/ Poor
5. Sample Collected: Oral swab / Rectal swab / Urine swab

**Section D: Morphometric and demographic characteristics (Researcher only)**

1. Body weight (g): _______________
2. Body length (snout to base of tail, cm): _______________
3. Tail length (cm): _______________
4. Ear length (cm): _______________
5. Health condition: Apparently healthy / sick/ Diseased
6. Visible wounds or lesions on body?
7. Yes → Location: ______________
8. No
